# Chronic Stress Promotes Gastric Cancer Metastasis by Inducing Microbiota‐Associated Kynurenine Accumulation and Macrophage Remodeling

**DOI:** 10.1002/advs.77612

**Published:** 2026-09-03

**Authors:** Sicheng Zhao, Zhiyuan Song, Jingcheng Zhang, Jiaheng Lou, Shuo Zhang, Boqiang Liu, Yunhai Wei, Fengmei Qiu, Tao Jiang, Guangji Zhang

**Affiliations:** ^1^ School of Basic Medical Sciences Zhejiang Chinese Medical University Hangzhou China; ^2^ Key Laboratory of Blood‐Stasis‐Toxin Syndrome of Zhejiang Province Hangzhou China; ^3^ Department of General Surgery Sir Run Run Shaw Hospital School of Medicine Zhejiang University Hangzhou China; ^4^ National Engineering Research Center of Innovation and Application of Minimally Invasive Instruments Hangzhou China; ^5^ Zhejiang Minimal Invasive Diagnosis and Treatment Technology Research Center of Severe Hepatobiliary Disease Sir Run Run Shaw Hospital School of Medicine Zhejiang University Hangzhou China; ^6^ Huzhou Central Hospital Fifth School of Clinical Medicine of Zhejiang Chinese Medical University Huzhou China; ^7^ Huzhou Central Hospital Affiliated Central Hospital of Huzhou University Huzhou China; ^8^ School of Pharmaceutical Sciences Zhejiang Chinese Medical University Hangzhou China; ^9^ Traditional Chinese Medicine “Preventing Disease” Wisdom Health Project Research Center of Zhejiang Hangzhou China

**Keywords:** C/EBPβ, chronic stress, gastric cancer, gut microbiota, kynurenine, *Lactobacillus reuteri*, metastasis, TNFR2, tumor‐associated macrophages

## Abstract

Chronic stress frequently occurs in patients with cancer, yet how stress is translated into pro‐metastatic programs remains unclear. Here, using chronic restraint stress (CRS) in male mice, we show that stress increases metastatic burden and reduces survival in lung metastasis and peritoneal dissemination models, while also increasing overall tumor burden in an orthotopic gastric cancer model. Single‐cell transcriptomics of lung metastases reveals stress‐associated remodeling of tumor‐associated macrophages toward immunosuppressive states, while macrophage depletion attenuates stress‐enhanced metastasis. Stress also reshapes the gut microbiota and enriches *Lactobacillus reuteri*, and microbiota transfer, antibiotic treatment, co‐housing, and bacterial gavage experiments support a microbiota‐dependent contribution to metastatic progression. Metabolomic analyses identify kynurenine as a stress‐ and *L. reuteri*‐associated tryptophan metabolite that promotes macrophage‐dependent dissemination, whereas tryptophan deficiency blunts stress‐ and *L. reuteri*‐enhanced metastasis. Mechanistically, our data support a role for TNFR2–C/EBPβ signaling in Kyn‐induced immunosuppressive macrophage remodeling. In clinical gastric cancer cohorts, higher depressive symptom burden is associated with shorter disease‐free survival, *Lactobacillus* enrichment, elevated plasma Kyn/Trp ratio, and altered macrophage phenotypes. Together, these findings define a stress–microbiota–tryptophan metabolism–macrophage axis that links chronic stress to gastric cancer metastasis in male mouse models and clinical samples.

## Graphical abstract

Chronic stress reshapes the gut microbiota and enriches Lactobacillus reuteri, promoting microbiota‐associated kynurenine accumulation through altered tryptophan metabolism. Kynurenine drives immunosuppressive remodeling of tumor‐associated macrophages through TNFR2–C/EBPβ signaling, thereby enhancing gastric cancer dissemination. This stress–microbiota–metabolism–macrophage axis links psychological stress to metastatic progression.

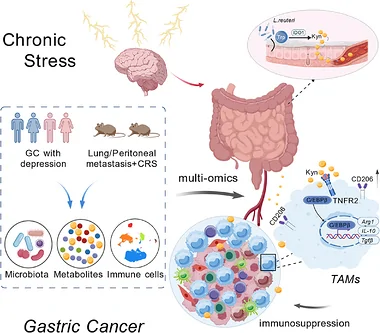

## 1 Introduction

Gastric cancer continues to impose a substantial global mortality burden [1], with metastatic disease accounting for much of this burden [2]. Despite progress in surgical management and systemic treatment, the prognosis of patients with metastatic gastric cancer continues to be unfavorable [3, 4], underscoring the need to identify major determinants of metastatic progression. Growing evidence suggests that psychosocial factors, especially chronic stress, substantially influence cancer progression, patient survival, and responses to systemic anticancer therapy [5, 6, 7]. However, the biological mechanisms through which chronic stress facilitates gastric cancer metastasis remain incompletely understood.

Stress responses are largely governed by activation of the hypothalamic–pituitary–adrenal axis and the sympathetic nervous system, leading to persistent increases in glucocorticoids and catecholamines [8]. These neuroendocrine mediators can reshape the tumor microenvironment and promote tumor progression, partly through attenuation of antitumor immunity [9]. Within the tumor microenvironment, macrophages constitute a major immune cell population with considerable phenotypic and functional heterogeneity [10, 11]. Available evidence indicates that macrophage polarization toward an immunosuppressive state contributes substantially to tumor dissemination and immune escape [12, 13, 14], while findings from diverse tumor models suggest that chronic stress can shift macrophage programs toward immunosuppressive states, thereby accelerating tumor progression [12, 15].

Changes in the gut microbiota have been associated with cancer progression, therapeutic resistance, and immune regulation in gastrointestinal malignancies [16, 17, 18], highlighting the importance of host–microbiota interactions in tumor biology. In addition to direct neuroendocrine–immune crosstalk, emerging studies indicate that the gut microbiota may transmit or modulate stress‐related influences on tumor progression [7, 19]; however, the contribution of this stress–microbiota axis to gastric cancer metastasis has not been fully resolved.

Mechanistically, microbial metabolites serve as an important interface between alterations in the gut microbiota and host immune regulation. Tryptophan metabolism is a major metabolic route through which gut microbes influence immune function [20, 21]. The kynurenine pathway, in particular, has been implicated in immune tolerance and tumor progression by modulating immune cell differentiation and function [22, 23, 24]. Increased kynurenine concentrations have been reported across multiple cancer types and are linked to immunosuppressive macrophage polarization and adverse clinical outcomes [25, 26], stimulating efforts to reduce kynurenine levels therapeutically through kynureninase‐based approaches [27].

Here, we demonstrate that chronic stress enhances gastric cancer metastasis and reduces survival in a microbiota‐dependent fashion. Chronic stress produces gut microbial dysbiosis accompanied by enrichment of *Lactobacillus reuteri*, which subsequently alters host tryptophan metabolism and increases systemic kynurenine levels. Kynurenine promotes macrophage remodeling toward an immunosuppressive state, while our findings implicate TNFR2–C/EBPβ signaling in this process. Collectively, our results identify a previously unrecognized axis connecting chronic stress with gastric cancer metastasis and suggest potential therapeutic opportunities at the interface of microbial metabolism and macrophage signaling.

## 2 Results

### 2.1 Stress Increases Metastatic and Orthotopic Tumor Burden and Reduces Survival

**FIGURE 1:**
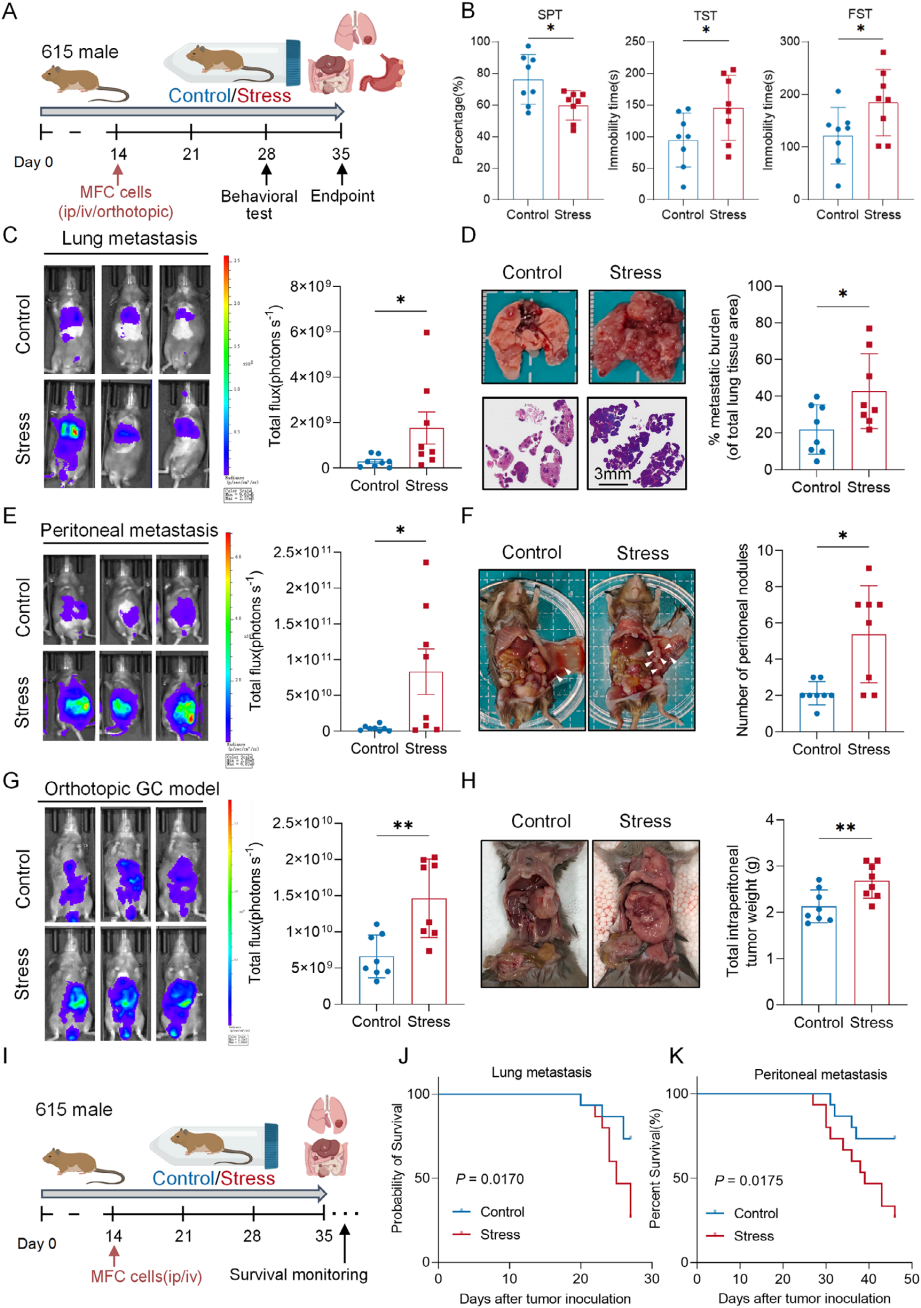
Chronic restraint stress increases metastatic and orthotopic tumor burden and reduces survival. (A) Design of the mouse experiments. (B) Behavioral phenotypes assessed by SPT, TST, and FST (*n* = 8). (C) Lung metastatic burden assessed by BLI, with total photon flux (*n* = 8). (D) Gross and H&E assessment of lung metastases, with metastatic area relative to total lung area (*n* = 8; scale bar = 3 mm). (E) Peritoneal dissemination assessed by BLI, with total photon flux (*n* = 8). (F) Peritoneal metastatic nodules and corresponding nodule counts (*n* = 8). (G) Orthotopic gastric tumor burden assessed by BLI, with total photon flux (*n* = 8). (H) Gross orthotopic tumor burden and total intraperitoneal tumor weight (*n* = 8). Total intraperitoneal tumor weight represents the combined weight of visible intraperitoneal tumor lesions collected at endpoint. (I–K) Design of the survival experiment (I) and Kaplan–Meier curves for lung metastasis (J) and peritoneal dissemination (K) under Control and CRS conditions (*n* = 15). Values are shown as mean ± SD. *P < 0.05, **P < 0.01; ns, not significant. Statistical tests: two‐tailed unpaired Student's t tests for B–H; log‐rank tests for J and K.

To determine the impact of chronic stress on gastric cancer progression across different experimental settings, male mice were subjected to a 5‐week chronic restraint stress regimen, and tumor cells were inoculated on day 14 to establish lung metastasis, peritoneal dissemination, and orthotopic gastric cancer models (Figure 1A). Behavioral assays confirmed successful stress induction: compared with Control mice, Stress mice had lower sucrose preference and increased immobility in both the tail suspension and forced swim tests (Figure 1B). Stress was also associated with lower body weight during the experimental period and higher plasma cortisol and norepinephrine levels (Figure S1A–D).

In vivo bioluminescence imaging and histological analyses showed that chronic stress increased lung metastatic burden, as reflected by higher photon flux and larger metastatic area (Figure 1C,D). Stress also enhanced peritoneal dissemination, with higher photon flux and more peritoneal metastatic nodules (Figure 1E,F). In the orthotopic gastric cancer model, chronic stress increased overall tumor burden, as indicated by stronger bioluminescence signals and greater total intraperitoneal tumor weight (Figure 1G,H). In an independent survival cohort (Figure 1I), chronic stress significantly reduced overall survival in both lung metastasis and peritoneal dissemination models (Figure 1J,K). In contrast, subcutaneous primary tumor growth was not significantly altered by stress (Figure S1E–G).

### 2.2 Single‐Cell Profiling Reveals Stress‐Associated Remodeling of Heterogeneous Macrophage States

**FIGURE 2:**
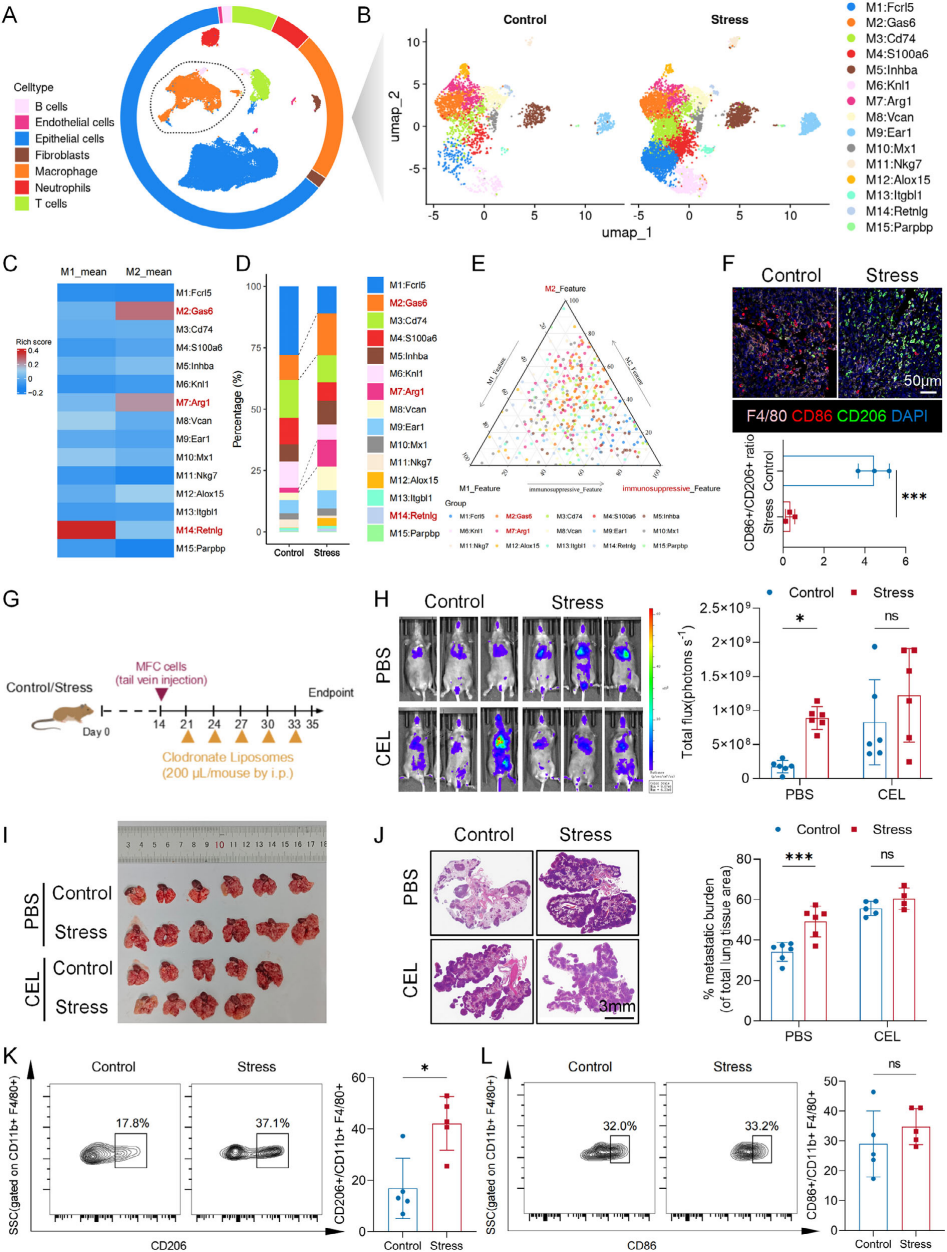
Single‐cell profiling identifies stress‐enriched immunosuppressive macrophage states and supports a macrophage contribution to metastatic progression. (A) scRNA‐seq overview of major immune and nonimmune populations in lung metastatic lesions. (B) UMAP of reclustered macrophages from Control and Stress mice. (C) M1‐ and M2‐associated signature scores across macrophage subsets. (D) Macrophage subset distribution between Control and Stress groups. (E) Ternary positioning of macrophage subsets according to M1‐associated, M2‐associated, and immunosuppressive signatures. (F) Immunofluorescence of lung metastatic lesions stained for F4/80, CD86, CD206, and DAPI, with CD86^+^/CD206^+^ macrophage ratios (*n* = 3 per group; scale bar = 50 µm). (G) Design of in vivo macrophage depletion with clodronate liposomes under Control or CRS conditions. (H) BLI‐based lung metastatic burden after PBS or clodronate‐liposome treatment, with total photon flux (*n* = 6 per group). (I) Gross lung appearance across the indicated groups. (J) H&E‐based lung metastatic burden, expressed as metastatic area relative to total lung area. Only lungs collected at the predefined endpoint without excessive residual blood were included; sample sizes are indicated by individual data points. (K, L) CD206^+^ (K) and CD86^+^ (L) macrophage frequencies among tumor‐infiltrating macrophages in Control and Stress mice (*n* = 6). Values are shown as mean ± SD. *P < 0.05, ***P < 0.001; ns, not significant. Statistical tests: two‐way ANOVA with Sidak's multiple‐comparisons test for H and J; two‐tailed unpaired Student's t tests for K and L.

To identify the cellular populations associated with stress‐enhanced metastasis, we performed scRNA‐seq of lung metastatic lesions. Unsupervised clustering identified seven major cell populations across immune and nonimmune compartments in both groups (Figure 2A; Figure S2A). Macrophages, the predominant immune population within metastatic lesions (Figure S2B), were prioritized for further characterization because of their abundance and functional plasticity.

Reclustering identified 15 macrophage subsets with distinct transcriptional states (Figure 2B). Heatmap analysis based on canonical M1‐ and M2‐associated genes revealed pronounced heterogeneity across macrophage subsets, particularly in the expression of pro‐inflammatory genes and immunosuppressive genes (Figure S2C). To provide a simplified functional reference for these heterogeneous macrophage states, M1‐ and M2‐associated signature scores were calculated and compared across macrophage subsets to assess their inflammatory and immunosuppressive tendencies (Figure 2C). Projection of these signature scores onto the UMAP space revealed a continuous spectrum between M1‐ and M2‐associated states rather than discrete polarization (Figure S2D).

Notably, macrophage cluster M2 (Gas6) and cluster M7 (Arg1) were preferentially enriched in Stress mice and exhibited tissue‐remodeling transcriptional features. Using the M1/M2‐associated signatures as simplified functional references, these stress‐enriched subsets showed a stronger tendency toward conventional M2‐associated and immunosuppressive features (Figure 2D). This tendency was further supported by ternary plot analysis (Figure 2E). In line with these findings, qPCR analysis of lung metastatic lesions revealed upregulation of genes commonly associated with immunosuppressive and M2‐like macrophage programs, including *Arg1*, *Mrc1*, *Tgfb1*, and *Il10*, together with reduced *Il1b* expression in Stress mice (Figure S2E). IHC and immunofluorescence further supported this phenotype, showing stronger ARG1 staining and an increased abundance of F4/80^+^ CD206^+^ macrophages, respectively, in metastatic lesions from Stress mice (Figure S2F; Figure 2F). At the systemic level, ELISA showed increased circulating IL‐10 and TGFβ in Stress mice (Figure S2G,H).

To further evaluate the functional contribution of macrophages to stress‐enhanced metastasis, we next performed in vivo macrophage depletion using clodronate liposomes (Figure 2G). Flow cytometry confirmed effective depletion of macrophages in both tumor and spleen tissues (Figure S2I). Under macrophage‐depleted conditions, the difference in metastatic burden between Stress and Control mice was markedly attenuated (Figure 2H–J). In parallel, under non‐depleted conditions, Stress mice displayed a higher proportion of CD206^+^ macrophages, whereas the proportion of CD86^+^ macrophages remained largely unchanged (Figure 2K,L). By contrast, the proportions of CD4^+^ T cells, CD8^+^ T cells, NK cells, and regulatory T cells were comparable between groups (Figure S2J).

### 2.3 Stress‐Associated Gut Dysbiosis Drives Metastatic Progression in a Microbiota‐Dependent Manner

To assess the contribution of gut microbial changes to stress‐associated metastasis, fecal microbiota from Control and Stress mice were profiled by 16S rRNA gene sequencing. β‐diversity analysis by principal coordinates analysis (PCoA) revealed distinct community structures between the two groups (Figure S3A), together with lower α‐diversity and a higher microbiota dysbiosis index (MDI) in Stress mice (Figure S3B,C). Shared and unique OTUs were further summarized (Figure S3D), and taxonomic profiling revealed stress‐associated shifts at both the family and genus levels (Figure S3E,F).

**FIGURE 3:**
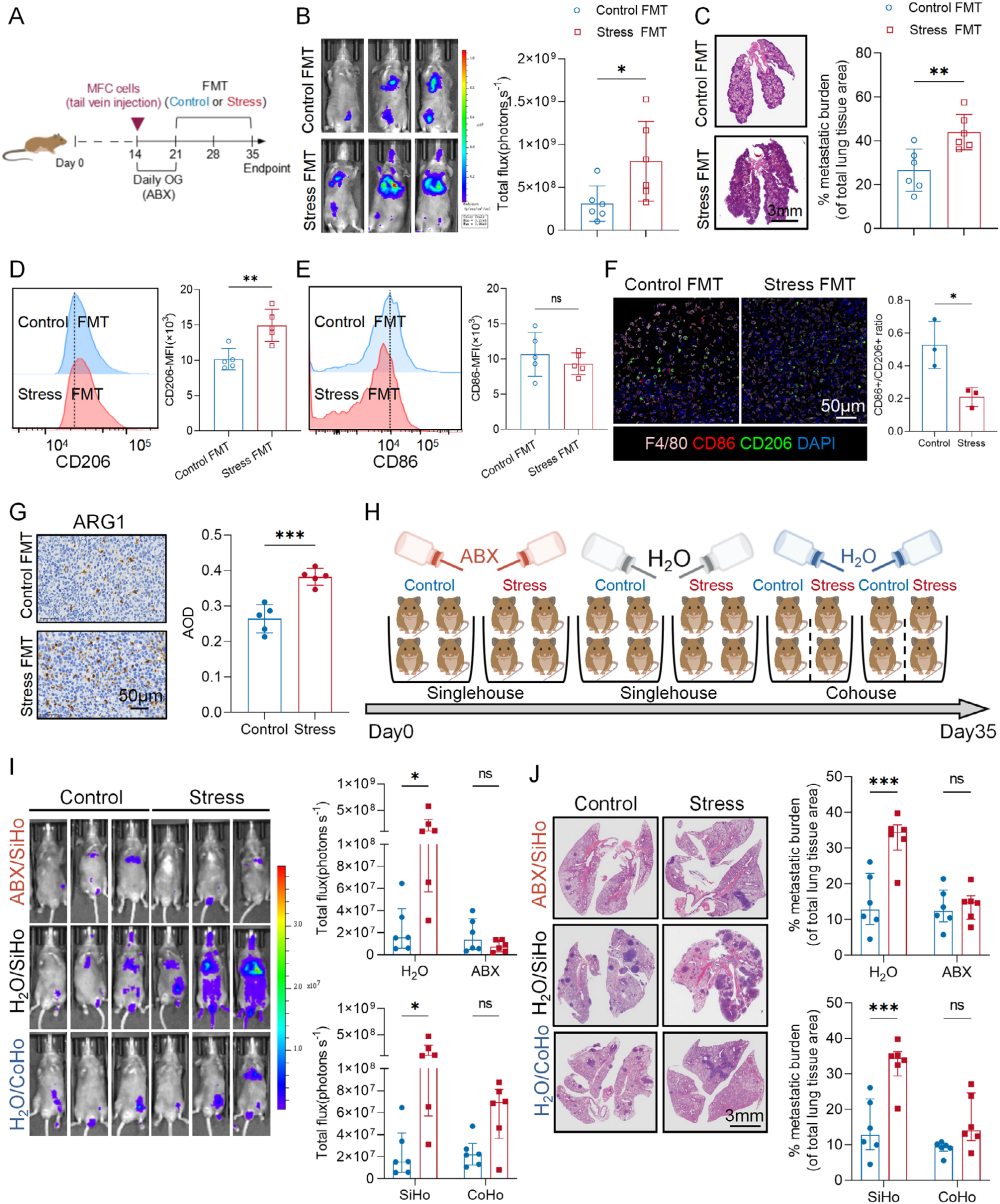
Stress‐associated gut dysbiosis drives metastatic progression in a microbiota‐dependent manner. (A) Design of the fecal microbiota transplantation (FMT) experiment. (B) Lung metastatic burden by BLI in recipients of Control‐ or Stress‐derived microbiota, with total photon flux (*n* = 6). (C) H&E assessment of lung metastatic burden in Control‐FMT and Stress‐FMT recipients (*n* = 6; scale bar = 3 mm). (D, E) CD206 (D) and CD86 (E) MFI in CD11b^+^F4/80^+^ macrophages from metastatic lungs after FMT (*n* = 6). (F) Immunofluorescence of lung metastatic lesions stained for F4/80, CD86, CD206, and DAPI, with corresponding CD86^+^/CD206^+^ macrophage ratios (*n* = 3 per group; scale bar = 50 µm). (G) ARG1 immunohistochemistry in lung metastatic lesions with AOD quantification (*n* = 6; scale bar = 50 µm). (H) Design of the antibiotic‐depletion and co‐housing experiments used to evaluate microbiota dependence. (I) BLI‐based lung metastatic burden under the indicated conditions, with total photon flux (*n* = 6). (J) H&E‐based lung metastatic burden under the same conditions (*n* = 6; scale bar = 3 mm). Values are shown as mean ± SD. *P < 0.05, **P < 0.01, ***P < 0.001; ns, not significant. Statistical tests: two‐tailed unpaired Student's t tests for B–E and G; two‐way ANOVA with Sidak's multiple‐comparisons test for I and J.

To test whether stress‐associated microbiota are sufficient to transfer the pro‐metastatic phenotype, we performed fecal microbiota transplantation (FMT) (Figure 3A). Antibiotic pretreatment efficiently depleted endogenous microbiota (Figure S3G). Recipients of microbiota from Stress donors developed a greater metastatic burden than Control FMT recipients (Figure 3B,C). 16S profiling of recipient mice confirmed a divergence in microbial community structure and MDI between the two FMT groups (Figure S3H,I). At the immune level, Stress FMT increased CD206 mean fluorescence intensity in CD11b^+^ F4/80^+^ macrophages within lung metastatic lesions, whereas CD86 mean fluorescence intensity remained comparable between groups (Figure 3D,E). Consistently, immunofluorescence confirmed increased CD206 signal in F4/80^+^ macrophages (Figure 3F), and IHC showed stronger ARG1 staining in metastatic lesions from Stress FMT recipients (Figure 3G). Circulating IL‐10 levels were also elevated in Stress FMT recipients (Figure S3J).

To further test whether the pro‐metastatic effect of stress is microbiota‐dependent, we employed two complementary approaches: broad‐spectrum antibiotic treatment to deplete the gut microbiota and co‐housing to equalize microbial exposure between Control and Stress mice (Figure 3H). During antibiotic treatment, fecal bacterial DNA levels were markedly reduced (Figure S3K), and under these conditions the stress‐associated increase in metastatic burden was attenuated (Figure 3I,J). Likewise, co‐housing diminished differences in metastatic progression between Control and Stress mice (Figure 3I,J), promoted convergence of gut microbial community structures (Figure S3L), and was accompanied by corresponding changes in α‐diversity (Figure S3M). Notably, co‐housing also partially restored body weight relative to single‐housed stressed mice (Figure S3N).

### 2.4 Stress‐Enriched *Lactobacillus reuteri* Promotes Tryptophan‐Dependent Metastasis

**FIGURE 4:**
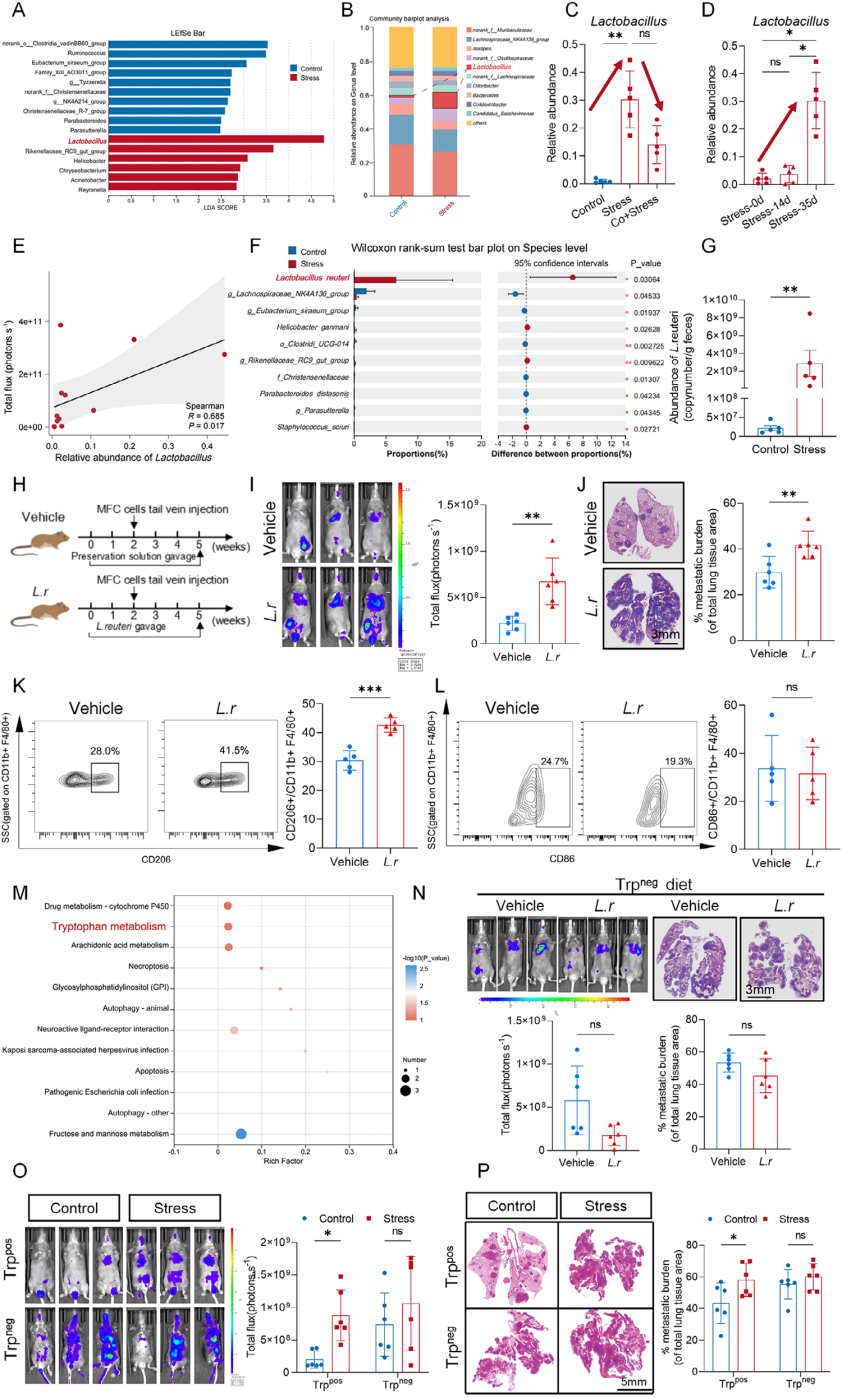
Stress‐enriched *Lactobacillus reuteri* promotes tryptophan‐dependent metastasis. (A) LEfSe‐defined bacterial taxa differing between Control and Stress mice. (B) Genus‐level fecal microbiota composition in Control and Stress groups. (C) Fecal *Lactobacillus* abundance in Control, Stress, and co‐housed mice before endpoint (*n* = 5). (D) Longitudinal fecal *Lactobacillus* abundance during stress exposure (*n* = 5). (E) Association between fecal *Lactobacillus* abundance and tumor bioluminescence intensity. (F) Species‐level differences between Control and Stress mice, highlighting enrichment of *L. reuteri*. (G) Absolute fecal *L. reuteri* abundance in Control and Stress mice (*n* = 5). (H) Design of the *L. reuteri* gavage experiment. (I) Lung metastatic burden by BLI after Vehicle or *L. reuteri* treatment, with total photon flux (*n* = 6). (J) H&E assessment of lung metastatic burden after Vehicle or *L. reuteri* treatment (*n* = 6; scale bar = 3 mm). (K, L) Frequencies of CD206^+^ (K) and CD86^+^ (L) macrophages within CD11b^+^F4/80^+^ cells after Vehicle or *L. reuteri* treatment (*n* = 5). (M) Plasma metabolite pathway enrichment highlighting tryptophan metabolism. (N) Lung metastatic burden under tryptophan‐deficient conditions assessed by BLI and H&E (*n* = 6; scale bar = 3 mm). (O) BLI‐based lung metastatic burden in Control and Stress mice maintained on Trp^pos^ or Trp^neg^ diets (*n* = 6). (P) H&E‐based lung metastatic burden under the same dietary conditions (*n* = 6; scale bar = 3 mm). Values are shown as mean ± SD. *P < 0.05, **P < 0.01, ***P < 0.001; ns, not significant. Statistical tests: two‐tailed unpaired Student's t tests for G, I, J, K, L, and N; one‐way ANOVA with appropriate multiple‐comparisons testing for C and D; two‐way ANOVA with Sidak's multiple‐comparisons test for O and P.

To identify bacterial taxa potentially involved in stress‐associated metastasis, differential microbiota analysis using LEfSe highlighted enrichment of the genus *Lactobacillus* in Stress mice relative to Control mice (Figure 4A). This was also reflected in genus‐level community composition profiles, which showed increased relative abundance of *Lactobacillus* under stress (Figure 4B). In the co‐housing model, fecal *Lactobacillus* abundance was reduced in Stress mice compared with single‐housed Stress mice (Figure 4C), whereas longitudinal analysis showed a progressive expansion of *Lactobacillus* with prolonged stress exposure (Figure 4D). Importantly, across Control and Stress mice, *Lactobacillus* abundance positively correlated with tumor bioluminescence intensity (Figure 4E).

Species‐level analysis identified *Lactobacillus reuteri* as enriched in Stress mice (Figure 4F), and this finding was validated by qPCR using both relative and absolute quantification approaches (Figure 4G; Figure S4A). We next evaluated the effect of *L. reuteri* on metastatic progression by sustained oral gavage. This treatment increased fecal *L. reuteri* abundance at the experimental endpoint (Figure S4C), without significantly affecting body weight during treatment (Figure S4B), and resulted in a greater metastatic burden (Figure 4I,J). In parallel, *L. reuteri* treatment increased the proportion of CD206^+^ but not CD86^+^ macrophages (Figure 4K,L), and was accompanied by a greater abundance of F4/80^+^ CD206^+^ macrophages in metastatic lesions (Figure S4D), showing a pattern similar to that observed under stress.

Given that gut microbiota can influence host physiology through metabolic remodeling, we conducted untargeted plasma metabolomic profiling, which revealed clear metabolic alterations under stress (Figure S4E). Pathway enrichment analysis identified tryptophan metabolism as one of the most significantly altered metabolic pathways (Figure 4M). In light of the reported link between *Lactobacillus* and host tryptophan metabolism [28, 29, 30], we fed mice a tryptophan‐deficient diet to test whether tryptophan availability is required for the tumor‐promoting effect of *L. reuteri* (Figure S4F). Under these conditions, *L. reuteri* administration no longer enhanced metastasis (Figure 4N). Chronic stress increased lung metastatic burden under a tryptophan‐containing diet (Figure S4G), whereas no significant difference was observed between Control and Stress mice under a tryptophan‐deficient diet (Figure 4O,P), suggesting that chronic stress‐enhanced metastasis is tryptophan‐dependent.

To determine whether the effect of *L. reuteri* might vary across tumor contexts, we further tested the same gavage regimen in a subcutaneous model (Figure S4H). In this setting, *L. reuteri* treatment reduced tumor growth (Figure S4I,J), accompanied by differences in immune composition relative to lung metastatic lesions, including a lower CD45^+^ fraction and a higher Ly6C^+^ fraction within the CD11b^+^ F4/80^+^ compartment (Figure S4K,L).

### 2.5 Kynurenine Accumulates Under Stress and Promotes Metastasis via Macrophages

**FIGURE 5:**
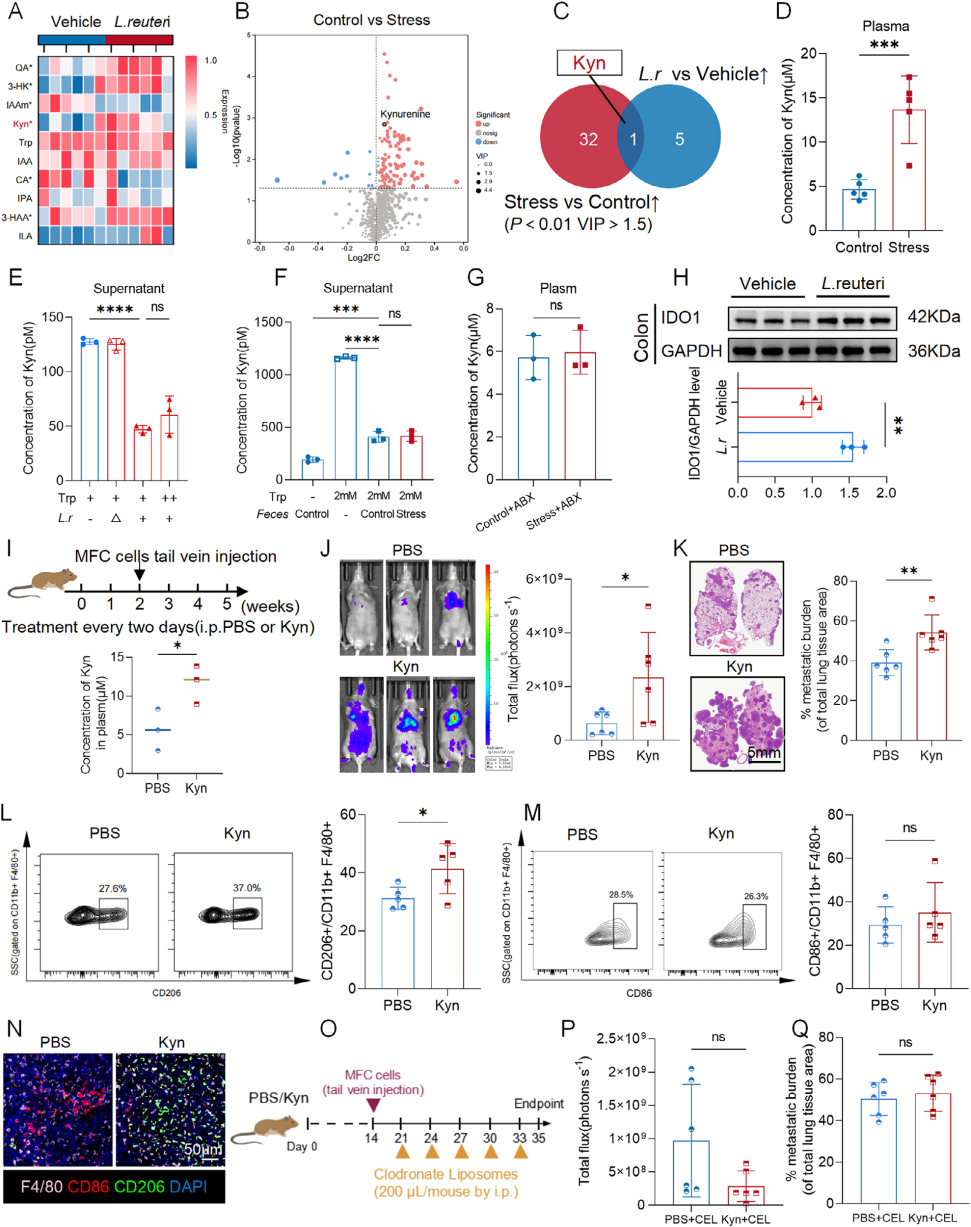
Stress‐associated kynurenine promotes metastasis through immunosuppressive macrophage remodeling. (A) Targeted tryptophan‐pathway metabolite profiles across the indicated groups. (B) Differential plasma metabolites identified by untargeted metabolomics in Control versus Stress mice. (C) Overlap between stress‐associated differential metabolites (*P* < 0.01, VIP > 1.5) and metabolites increased after *L. reuteri* treatment in targeted metabolomics. (D) Plasma Kyn concentrations in Control and Stress mice (*n* = 5). (E) Kyn concentrations in bacterial culture supernatants (*n* = 3). (F) Kyn concentrations in fecal‐community culture supernatants (*n* = 3). (G) Plasma Kyn concentrations after ABX treatment in Control and Stress mice (*n* = 3). (H) IDO1 protein levels in colon tissues after Vehicle or *L. reuteri* treatment, with IDO1/GAPDH quantification (*n* = 3). (I) Design of the in vivo Kyn‐treatment experiment, with plasma Kyn concentrations measured before endpoint (*n* = 3). (J) Lung metastatic burden assessed by BLI after PBS or Kyn treatment, with total photon flux (*n* = 6). (K) H&E assessment of lung metastatic burden after PBS or Kyn treatment (*n* = 6; scale bar = 5 mm). (L, M) CD206^+^ (L) and CD86^+^ (M) macrophage frequencies within CD11b^+^F4/80^+^ cells after PBS or Kyn treatment (*n* = 5). (N) Immunofluorescence of lung metastatic lesions stained for F4/80, CD86, CD206, and DAPI (scale bar = 50 µm). (O) Design of macrophage depletion during Kyn treatment. (P) Total photon flux after clodronate‐liposome treatment in PBS and Kyn groups (*n* = 6). (Q) Lung metastatic burden after clodronate‐liposome treatment in PBS and Kyn groups (*n* = 6). Values are shown as mean ± SD. *P < 0.05, **P < 0.01, ***P < 0.001, ****P < 0.0001; ns, not significant. Statistical tests: two‐tailed unpaired Student's t tests for D, G–M, P, and Q; one‐way ANOVA with Tukey's multiple‐comparisons test for E and F.

To identify the metabolite driving *L. reuteri*–associated metastasis, we performed targeted profiling of tryptophan metabolites in plasma from the *L. reuteri* gavage model. Global analysis revealed a distinct metabolic profile compared with vehicle‐treated mice (Figure S5A). Within the kynurenine pathway, several metabolites, including kynurenine (Kyn), 3‐hydroxykynurenine (3‐HKYN), and quinolinic acid (QA), were significantly elevated in *L. reuteri*–treated mice (Figure 5A). We then intersected these changes with the stress‐associated untargeted metabolomic dataset (Figure 5B), which identified Kyn as the only metabolite consistently elevated in both contexts (Figure 5C). ELISA further showed that Kyn concentrations were increased in plasma, feces, and tumor tissues from Stress mice (Figure 5D; Figure S5B,C). Moreover, in both the stress model and the *L. reuteri* gavage model, Kyn levels positively correlated with metastatic burden (Figure S5D,E).

To clarify the source of Kyn accumulation, we first tested whether Kyn could be directly produced by *L. reuteri*. Bacteria were cultured in tryptophan‐supplemented medium (Figure S5F), and Kyn concentrations were found to be lower in supernatants from live *L. reuter*i cultures than in medium‐only controls or heat‐killed cultures (Figure 5E), arguing against direct Kyn production by *L. reuteri* under our culture conditions. Instead, live *L. reuteri* reduced Kyn levels in the medium. Because microbial metabolic outputs can arise from community‐level cooperation [31], we next performed ex vivo fecal community cultures (Figure S5F). However, communities derived from Stress mice did not exhibit a greater capacity to generate Kyn than those from Control mice (Figure 5F). We therefore asked whether stress‐associated Kyn elevation was dependent on the gut microbiota. Under antibiotic‐treated conditions, the stress‐associated systemic increase in Kyn was no longer significant (Figure 5G; Figure S5G,H). Considering that gut microbes could regulate host tryptophan metabolism [20, 32, 33], we examined host enzymes involved in Kyn biosynthesis (Figure S5I) and observed increased IDO1 expression in the colon (Figure 5H), suggesting that microbiota‐associated signals may contribute to Kyn accumulation via host Trp metabolism. In addition, we observed decreased expression of intestinal barrier proteins in the bacteria‐treated group, indicating that bacterial colonization may also impair gut barrier integrity (Figure S5J).

We next examined whether Kyn is functionally sufficient to promote metastatic progression. Across the tested concentrations, Kyn had minimal impact on gastric cancer cell clonogenicity and motility (Figure S5K,L). By contrast, Kyn induced an M2‐like program in macrophages, increasing M2‐associated gene expression and the CD206^+^ fraction in BMDMs and RAW264.7 cells (Figure S5M,N). In vivo, intraperitoneal injection of Kyn increased circulating Kyn levels (Figure 5I), promoted tumor dissemination (Figure 5J,K), and was accompanied by a higher proportion of CD206^+^ TAMs in tumor tissues (Figure 5L–N). Finally, macrophage depletion with clodronate liposomes (Figure 5O) markedly blunted the pro‐metastatic effect of Kyn (Figure 5P,Q), supporting macrophages as key mediators of Kyn‐driven dissemination.

### 2.6 TNFR2–C/EBPβ Signaling Is Implicated in Stress‐Associated Macrophage Remodeling and Metastatic Progression

To dissect how stress‐associated Kyn drives macrophage phenotypic modulation, we first performed bulk RNA‐seq of Kyn‐treated BMDMs. Given prior reports linking Kyn to AHR signaling, we initially performed GSEA, which revealed enrichment of an AHR response signature (Figure S6A). However, pharmacologic AHR antagonism with SR‐1 did not attenuate the Kyn‐induced increase in CD206 signal (Figure S6B), despite evidence of effective AHR blockade (Figure S6C). These findings suggest that AHR signaling is unlikely to be the principal pathway mediating the effect of Kyn on TAMs under our experimental conditions.

**FIGURE 6:**
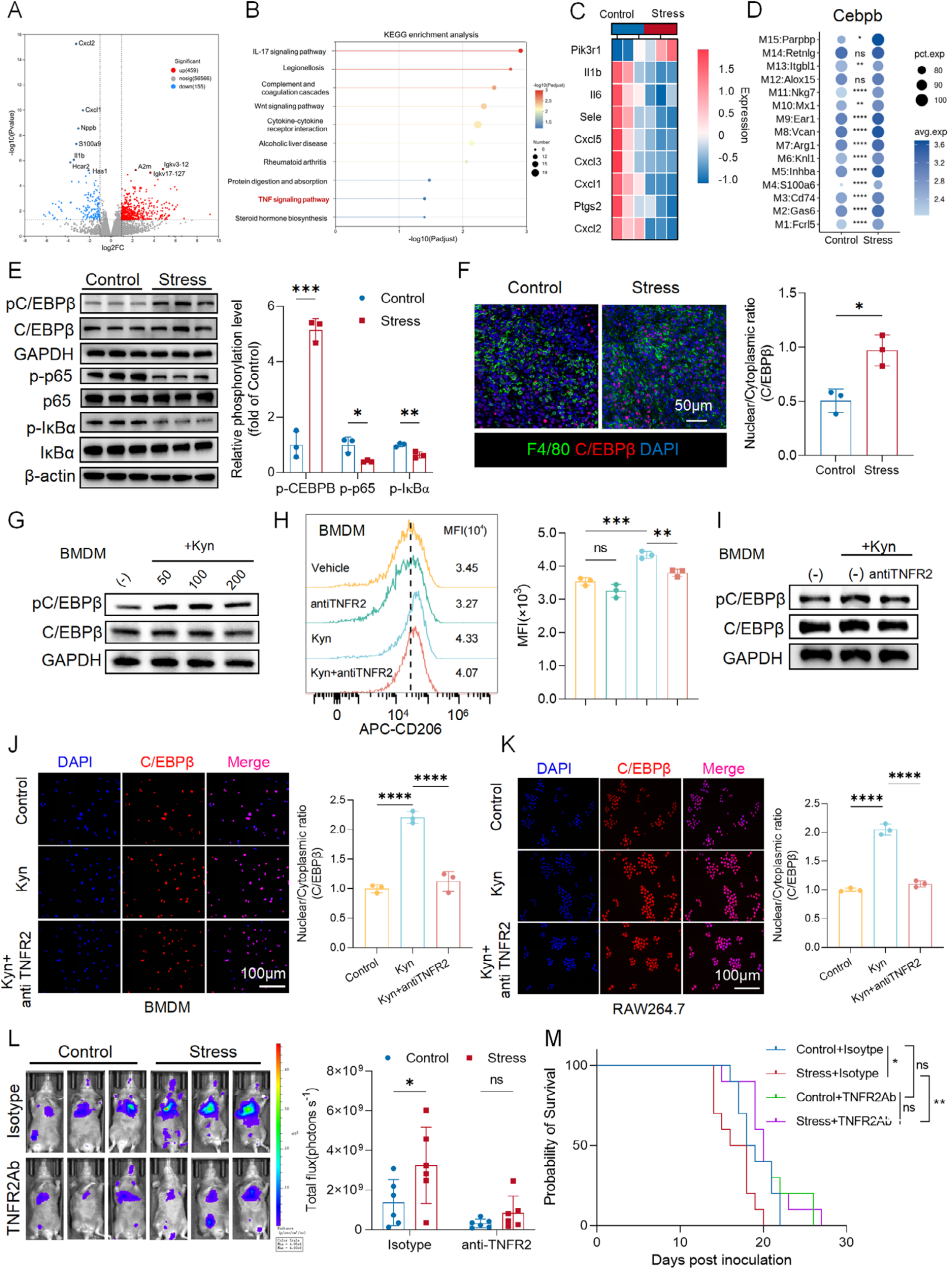
TNFR2–C/EBPβ signaling is implicated in Kyn‐induced immunosuppressive macrophage remodeling. (A) Differential gene expression in lung metastatic tissues from Control and Stress mice by bulk RNA‐seq. (B) KEGG enrichment of the differentially expressed genes in (A). (C) Expression heatmap of TNF pathway‐associated genes in Control and Stress groups. (D) Cebpb expression across macrophage subsets. (E) p‐C/EBPβ, C/EBPβ, p‐p65, p65, p‐IκBα, and IκBα protein levels in metastatic tumor tissues, with phosphorylation ratios quantified below (*n* = 3). (F) C/EBPβ localization in F4/80^+^ macrophages within lung metastatic lesions, with nuclear/cytoplasmic C/EBPβ ratios (*n* = 3; scale bar = 50 µm). (G) p‐C/EBPβ and total C/EBPβ levels in BMDMs exposed to the indicated Kyn concentrations. (H) CD206 MFI in BMDMs under the indicated treatments (*n* = 3). (I) p‐C/EBPβ and total C/EBPβ levels in BMDMs under the indicated conditions. (J, K) C/EBPβ localization in BMDMs (J) and RAW264.7 cells (K), with corresponding nuclear/cytoplasmic ratios (*n* = 3; scale bar = 100 µm). (L) Lung metastatic burden assessed by BLI, with corresponding total photon flux (*n* = 6). (M) Kaplan–Meier survival curves for the indicated groups (*n* = 10). Values are shown as mean ± SD. *P < 0.05, **P < 0.01, ***P < 0.001; ns, not significant. Statistical tests: multiple t tests for E; one‐way ANOVA with multiple‐comparisons correction for H, J, and K; two‐way ANOVA with Sidak's multiple‐comparisons test for L; log‐rank test for M.

Meanwhile, we performed bulk RNA‐seq of lung metastatic lesions from Control and Stress mice (Figure 6A), and KEGG enrichment analysis highlighted TNF‐related pathways (Figure 6B). Consistently, macrophage populations extracted from scRNA‐seq similarly identified enrichment of TNF signaling under stress (Figure S6D). Further inspection of TNF pathway–annotated differentially expressed genes showed reduced expression of multiple pro‐inflammatory mediators with *Pik3r1* relatively elevated in Stress lesions (Figure 6C). Given prior evidence [34] that TNFR2‐mediated signaling can engage PI3K‐associated immunoregulatory programs, suppress NF‐κB transcriptional activity, and promote C/EBPβ‐linked macrophage polarization, we next examined the TNFR2–C/EBPβ axis (Figure S6E).

Single‐cell analysis revealed increased Cebpb expression across most macrophage subsets under Stress, with a relative exception in the subset exhibiting higher M1‐associated features (Figure 6D). At the protein level, metastatic lung tissues from Stress mice showed elevated C/EBPβ phosphorylation, accompanied by reduced phosphorylation of NF‐κB–associated components (p‐p65 and p‐IκBα) (Figure 6E). Immunofluorescence further confirmed increased nuclear localization of C/EBPβ in F4/80^+^ macrophages within metastatic lesions (Figure 6F). Similarly, p‐C/EBPβ levels in metastatic lung tissue were elevated following *L. reuteri* gavage or Kyn treatment (Figure S6F,G). In contrast, the increase in p‐C/EBPβ induced by chronic stress was abrogated by antibiotic treatment or co‐housing (Figure S6H). Parallel immunofluorescence analysis further showed that stress‐induced C/EBPβ nuclear localization in F4/80^+^ macrophages was attenuated under antibiotic‐treated or co‐housed conditions (Figure S6I). These results indicate that gut microbiota contribute to stress‐induced C/EBPβ activation in the metastatic niche. Consistently, Kyn increased p‐C/EBPβ levels in BMDMs (Figure 6G).

To test whether TNFR2 signaling contributes to Kyn‐driven macrophage remodeling, we treated macrophages with a neutralizing anti‐TNFR2 antibody during Kyn stimulation. TNFR2 blockade attenuated Kyn‐induced immunosuppressive macrophage‐associated outputs, including reduced CD206 mean fluorescence intensity (Figure 6H), blunted induction of M2‐associated genes (Figure S6J), and reduced IL‐10 and TGFβ levels in the culture supernatant (Figure S6K). Consistent with these phenotypic changes, TNFR2 neutralization reduced Kyn‐induced C/EBPβ phosphorylation (Figure 6I) and diminished nuclear localization of C/EBPβ in BMDMs and RAW264.7 cells (Figure 6J,K). Pharmacologic inhibition of C/EBPβ with ST101 similarly mitigated Kyn‐induced CD206 upregulation (Figure S6L), supporting C/EBPβ as a functional downstream effector of this TNFR2‐dependent program. Finally, we evaluated TNFR2 blockade in vivo (Figure S6M) and found that TNFR2‐neutralizing antibody treatment reduced stress‐enhanced metastatic burden (Figure 6L) and improved survival relative to isotype‐treated stressed mice (Figure 6M).

Collectively, these findings support a role for TNFR2–C/EBPβ signaling in stress‐associated macrophage remodeling and metastatic progression.

### 2.7 Clinical Relevance of the Stress‐Associated Microbiota–Metabolism–Macrophage Axis in Gastric Cancer

**FIGURE 7:**
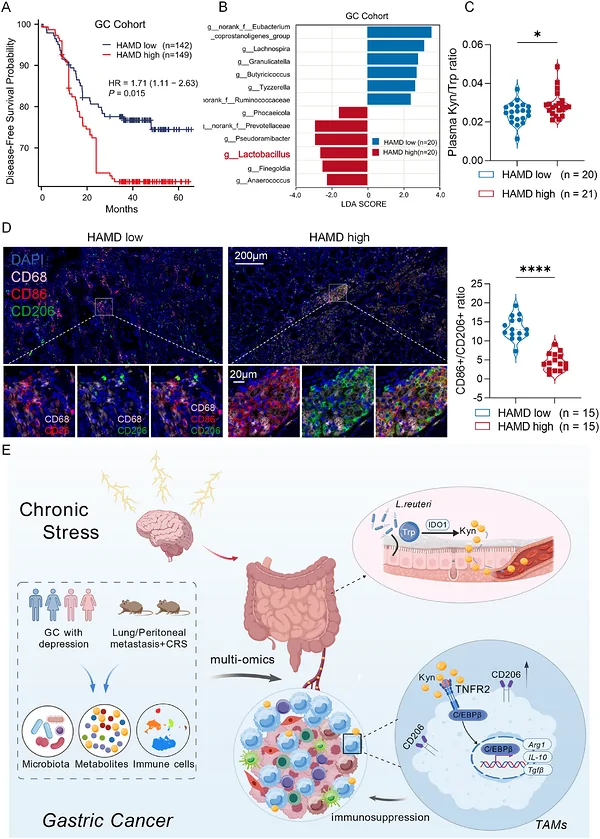
Clinical relevance and schematic model of the stress‐associated microbiota–metabolism–macrophage axis in GC. (A) Disease‐free survival according to HAMD status in patients with gastric cancer. (B) Differential bacterial taxa between HAMD‐low and HAMD‐high groups identified by LDA. (C) Plasma Kyn/Trp ratios in HAMD‐low and HAMD‐high patients, based on targeted tryptophan metabolomic profiling of 41 plasma samples. (D) Multiplex immunofluorescence of gastric cancer tissues stained for DAPI, CD68, CD86, and CD206, with corresponding CD68^+^CD86^+^/CD68^+^CD206^+^ ratios. Scale bars: 200 µm (upper) and 20 µm (lower). (E) Proposed model linking chronic stress to microbiota‐associated tryptophan metabolism, kynurenine accumulation, macrophage immunosuppressive remodeling, and metastatic progression, with TNFR2–C/EBPβ signaling implicated in the macrophage response. The schematic was created with BioGDP.com [35].

We further evaluated the clinical relevance of stress‐related alterations in gastric cancer patients. Patients with high HAMD scores showed significantly shorter disease‐free survival than those with low HAMD scores (Figure 7A). Microbial analysis revealed distinct gut microbiota profiles between the two groups, with *Lactobacillus* significantly enriched in the HAMD‐high group (Figure 7B). Targeted metabolomic profiling of available plasma samples further showed a significantly increased Kyn/Trp ratio in the HAMD‐high group (Figure 7C). In addition, multiplex IF staining showed a significant difference in the CD68^+^CD86^+^/CD68^+^CD206^+^ ratio between patients with low and high HAMD scores (Figure 7D). Together, these findings support the clinical relevance of a stress‐associated microbiota–metabolism–macrophage axis in gastric cancer (Figure 7E).

## 3 Discussion

Chronic stress is increasingly recognized as a modifier of cancer progression, yet how neuroendocrine disturbances are translated into pro‐metastatic programs remains incompletely understood [8, 36]. In this study, we show that chronic stress markedly increases GC metastatic burden in the lung metastasis and peritoneal dissemination models, underscoring a prominent role for stress in metastatic progression. Previous studies have implicated depression‐associated catecholamine/β2‐AR signaling in promoting GC dissemination in nude mice [37]. Our study establishes a chronic stress‐enhanced gastric cancer dissemination framework in immunocompetent male 615 mice, supported by experimental lung metastasis and peritoneal dissemination models, thereby providing a useful platform for dissecting immune mechanisms underlying stress‐driven metastatic progression.

TAMs, particularly those with M2‐like features, are well established as facilitators of gastric cancer dissemination [38, 39, 40], therapeutic resistance, and immune evasion [41, 42]. In this context, single‐cell profiling characterized stress‐associated immune remodeling in lung metastatic lesions and highlighted macrophages as a prominent immune population with marked stress‐associated changes. Notably, these macrophages exhibited marked heterogeneity and a continuum of states beyond a simple M1/M2 binary polarization model. Single‐cell profiling resolved stress‐associated macrophage heterogeneity and highlighted stress‐enriched macrophage states with immunosuppressive transcriptional features, which provided the basis for subsequent phenotypic validation and functional testing. These observations were supported by convergent evidence at the transcriptional, protein, and cytokine levels. Consistently, macrophage depletion attenuated stress‐enhanced metastatic burden, indicating a functional contribution of macrophages to stress‐driven metastatic progression. These findings extend prior observations that chronic stress can reshape myeloid programs toward immunosuppression and place this response in the setting of gastric cancer metastasis [12, 15].

Guided by group‐ and time‐resolved microbiota profiling, we identified *Lactobacillus* as a consistently stress‐enriched genus in our model [43, 44]. The significant association between *Lactobacillus* abundance and metastatic burden, together with species‐level enrichment of *Lactobacillus reuteri* under stress, prompted us to validate its pro‐metastatic effect in vivo. Notably, *L. reuteri* exerted divergent effects across tumor contexts, promoting metastatic progression in the lung dissemination model but suppressing tumor growth in the subcutaneous setting. Differences in the myeloid cell landscape across tumor models may partly account for the divergent effects observed across models. Although *Lactobacillus* species, including *L. reuteri*, are often regarded as beneficial probiotics, their biological effects appear to be highly context‐dependent. Recent studies have shown that *L. reuteri* or *Lactobacillus*‐derived metabolites can promote adverse tumor outcomes [45], regulate tumor‐associated macrophage immunosuppression through tryptophan metabolism [21], or contribute to treatment‐related intestinal injury in specific settings [46]. Together with our observation that *L. reuteri* promoted lung metastatic progression but suppressed subcutaneous tumor growth, these findings suggest that its effect is shaped by tumor niche, microbial metabolic output, and host immune‐metabolic context. It should be noted that the potential contributions of other microbial members or community‐level interactions cannot be excluded.

At the metabolic level, our data suggest that stress‐associated microbial remodeling promotes metastatic progression by reshaping host tryptophan–kynurenine metabolism and macrophage remodeling. Kyn is a well‐recognized immunoregulatory metabolite [47, 48, 49]. It has been implicated in chronic stress‐associated responses [44] and in the progression of gastric cancer [22, 23]. Here, Kyn was elevated in both the stress model and the *L. reuteri* gavage model, correlated with metastatic burden, and was sufficient to promote tumor dissemination in a macrophage‐dependent manner. Importantly, our data do not support *L. reuteri* as a direct producer of Kyn under the tested conditions. Although *L. reuteri* did not increase Kyn production in bacterial culture and Stress‐derived fecal microbial communities did not show enhanced Kyn‐generating capacity ex vivo, *L. reuteri* gavage increased colonic IDO1 expression, reduced intestinal barrier proteins, and elevated systemic Kyn levels by at least twofold. These findings suggest that *L. reuteri*‐associated Kyn accumulation may involve host‐mediated intestinal metabolic and barrier responses rather than direct bacterial synthesis alone. The elevated circulating Kyn may then act on macrophages within the lung metastatic niche to promote immunosuppressive phenotypic remodeling.

Integrating pathway enrichment from cell type‐resolved scRNA‐seq with independent bulk transcriptomics, we identified TNF‐related programs as a dominant stress‐associated signal. TNF signaling is a central immune axis, but its downstream outputs are highly receptor‐ and context‐dependent [50, 51]. Guided by prior work, we examined the TNFR2–C/EBPβ axis, which has been linked to immunosuppressive macrophage polarization [34, 52]. Prior studies suggest that diverse stress‐linked inputs—including adrenergic cues—can converge on C/EBPβ to shape myeloid programs [53, 54, 55]. Together, these findings point to a TNFR2–C/EBPβ module as a plausible conduit through which stress‐associated Kyn may drive macrophage remodeling.

This study has several limitations. First, all in vivo experiments were performed in male mice. Given that stress responses, HPA‐axis activity, immune regulation, and gut microbiome composition can differ between sexes, the present findings should be interpreted within the context of male mouse models. Future studies using female mice or sex‐comparative experimental designs will be important to determine whether the stress–microbiota–kynurenine–macrophage axis operates similarly across sexes. Second, although the lung metastasis and peritoneal dissemination models supported a pro‐metastatic effect of chronic stress, these experimental models do not fully recapitulate the spontaneous metastatic cascade arising from an orthotopic primary gastric tumor. In the current orthotopic model, metastatic burden was not evaluated using metastasis‐specific readouts, such as the incidence of peritoneal dissemination, the number of hepatic or pulmonary metastatic nodules, or histologically confirmed lymphatic spread. Consequently, the current assessment does not permit a rigorous characterization of the effect of chronic stress on spontaneous metastasis from an orthotopic gastric tumor, and further refinement of the model and its endpoint assessment is warranted. Third, although FMT, antibiotic treatment, and co‐housing supported the involvement of gut microbiota in stress‐enhanced metastatic progression and Kyn accumulation, these approaches remain indirect. Germ‐free mouse experiments would provide more definitive evidence for microbiota causality but were not feasible in the present study. Fourth, although we used species‐specific qPCR to quantify *Lactobacillus reuteri* in the mouse experiments, this targeted approach cannot define microbial functional genes involved in tryptophan metabolism, and our functional validation was limited to a single *L. reuteri* strain. In addition, the clinical 16S rRNA sequencing data support genus‐level enrichment of *Lactobacillus* but cannot definitively resolve species‐ or strain‐level changes. Future shotgun metagenomic studies using prospective cohorts and additional *L. reuteri* strains or clinical isolates will be needed to provide strain‐level and functional insight into microbiota‐associated metabolic regulation. Finally, genetic or cell type‐specific perturbation of key enzymes such as IDO1 would further strengthen the causal link between microbiota‐associated changes and host kynurenine‐pathway activation.

In summary, our findings support a stress–microbiota–kynurenine–TNFR2/C/EBPβ–macrophage axis that contributes to gastric cancer metastatic progression (Figure 7E). These findings provide a framework for understanding how chronic stress can be translated into immune and metabolic programs that support tumor dissemination, and they highlight the microbiota–metabolism–immune interface as a potential space for therapeutic intervention.

## 4 Experimental Model and Subject Details

### 4.1 Clinical Sample Collection

Clinical specimens were derived from gastric cancer cohorts recruited at Zhejiang Cancer Hospital, Zhejiang Provincial Hospital of Traditional Chinese Medicine, and Huzhou Central Hospital. The survival analysis comprised 291 patients. Separately, 40 fecal specimens from Zhejiang Cancer Hospital were analyzed under Institutional Review Board approval IRB‐2020‐25. Tumor tissues from 30 patients at Zhejiang Provincial Hospital of Traditional Chinese Medicine were used for multiplex immunostaining under ethics approval 2022‐KLS‐202‐02. For targeted tryptophan metabolomic profiling, plasma was obtained from 41 patients at Huzhou Central Hospital under ethics approval 20180701–07.

### 4.2 Mice

Male 615 mice (4 weeks old) were supplied by the Laboratory Animal Center of the Institute of Hematology, Chinese Academy of Medical Sciences. Animals were kept under SPF husbandry (22–26 °C; 45 ± 5% relative humidity; 12‐h light/dark cycle; 4–5 mice per cage) with unrestricted food and water access. Male C57BL/6 mice aged 6–8 weeks served as bone‐marrow donors for BMDM preparation. Animal use was authorized under protocol IACUC‐20230710‐03 by the ethics committee of Zhejiang Chinese Medical University.

### 4.3 Cell Lines

MFC‐LUC1 mouse gastric cancer cells were obtained from Zhejiang Meisen Cell Technology Co., Ltd. (Jinhua, China). HGC‐27 and AGS human gastric cancer cells and RAW264.7 murine macrophages were obtained from Seville Biotechnology Co., Ltd. (Wuhan, China). MFC‐LUC1 and HGC‐27 cells were maintained in RPMI‐1640, AGS cells in F‐12 medium, and RAW264.7 cells in DMEM, with all media supplemented with 10% fetal bovine serum. RAW264.7 cells (STCC20020G) were authenticated by short tandem repeat profiling.

### 4.4 Method Details

#### 4.4.1 Animal Tumor Models, Stress Paradigm, and In Vivo Interventions

All in vivo studies were conducted in 4‐week‐old male 615 mice. Following 3–5 d of acclimation, animals assigned to the CRS condition were confined individually in ventilated 50‐mL conical tubes from 08:00 to 16:00 each day for 35 d. During the corresponding 8‐h period, non‐stressed controls remained unrestrained but had food and water withheld to minimize intake‐related differences [7, 56].

For establishment of the subcutaneous tumor model, mice received a right‐flank injection of 1.5 × 10^6^ MFC‐LUC1 cells suspended in 100 µL PBS. Animals were euthanized on day 21 for plasma and tissue collection. Tumor burden remained below 2,000 mm^3^ in all mice.

For metastatic experiments, 1.5 × 10^6^ MFC‐LUC1 cells suspended in 100 µL PBS were administered on day 14. Lung dissemination was established by tail‐vein inoculation, whereas peritoneal dissemination was induced by intraperitoneal delivery of the same cell dose.

For the orthotopic gastric cancer model, MFC‐LUC1 cells were prepared as a single‐cell suspension at 1.5 × 10^6^ cells in 50 µL PBS. On day 14 after the start of the experimental schedule, mice were anesthetized, and the tumor cell suspension was injected into the gastric wall through a small upper abdominal incision. After confirming no obvious leakage, the stomach was repositioned in situ, followed by closure of the incision. At endpoint, visible intraperitoneal tumor lesions in the orthotopic model were carefully dissected and weighed together to calculate total intraperitoneal tumor weight.

Tumor burden was monitored by bioluminescence imaging on day 7 after tumor inoculation and again one day before sacrifice. Mice received intraperitoneal injection of luciferin substrate (CTCC‐Luc‐001, Meisen) at 100 µL/20 g body weight and were imaged 10 min later under 2% isoflurane anesthesia using an IVIS Lumina III system. Regions of interest were drawn over the chest or abdomen using Living Image software, and signal intensity was quantified as total flux (photons s^−1^).

For macrophage depletion experiments, clodronate liposomes (Yeasen, 40337ES10; 5 mg mL^−1^) were administered intraperitoneally at 25 mg kg^−1^ starting on day 21, followed by repeated injections every 3 d for a total of five injections. Control animals were given volume‐matched PBS at the corresponding injection time points.

For antibiotic cocktail‐mediated gut microbiota depletion, mice received a cocktail containing ampicillin (1 g L^−1^), metronidazole (1 g L^−1^), vancomycin (0.5 g L^−1^), and neomycin (0.5 g L^−1^), prepared in sterile drinking water and freshly replaced every 3 d. In experiments requiring gavage‐based pretreatment, the antibiotic cocktail was administered by oral gavage at 200 µL once daily, as indicated in the experimental design.

For fecal microbiota transplantation (FMT), the procedure was reported with reference to the GRAFT guidelines [57]. Fresh fecal pellets were collected from Control or Stress donor mice at the same time of day after spontaneous defecation, avoiding contamination with bedding or urine. Fecal samples from donor mice within the same group were pooled daily using approximately equal fecal weight from each donor and processed immediately. Briefly, feces were suspended in sterile ice‐cold 0.9% saline at a ratio of 1:10 (w/v) to obtain a fecal suspension of 100 mg mL^−1^. No long‐term storage or freeze–thaw cycles were used. Although the preparation was not performed in an anaerobic chamber, oxygen exposure was minimized by rapid processing on ice using pre‐chilled sterile materials, and the interval from fecal collection to oral gavage was kept within 30 min. After 10 s of vortexing, the fecal suspension was clarified by centrifugation at 800 × *g* for 3 min at 4 °C. The resulting supernatant was immediately used for FMT.

Recipient mice underwent microbiota depletion from day 14 through day 21 using daily oral administration of an antibiotic mixture containing ampicillin, metronidazole, vancomycin, and neomycin at final concentrations of 1, 1, 0.5, and 0.5 g L^−1^, respectively. Beginning on day 22, each mouse was gavaged once daily with 200 µL of freshly prepared fecal suspension until day 35. The inoculum was derived from Control donors for the Control‐FMT group and from Stress donors for the Stress‐FMT group. The effectiveness of microbiota depletion and subsequent FMT‐associated changes was evaluated by fecal bacterial culture, total fecal bacterial DNA quantification, and 16S rRNA gene sequencing.

For co‐housing experiments, control and stress mice were either maintained separately (three mice per cage) or co‐housed in mixed cages containing two control mice and two stress mice per cage during the experimental period, as indicated.

For *Lactobacillus reuteri* colonization experiments, *L. reuteri* (BNCC337178) was cultured anaerobically in MRS broth at 37 °C for 24–48 h and subcultured to ensure that bacteria used for animal experiments were within five passages. Log‐phase bacteria were collected, washed twice with sterile saline, and resuspended to 1 × 10^8^ CFU mL^−1^. Mice were gavaged daily with 200 µL bacterial suspension (2 × 10^7^ CFU per dose) for 5 weeks; control mice received an equal volume of sterile saline.

In the tryptophan‐restriction experiment, mice were maintained on a tryptophan‐deficient diet throughout the experimental period in place of standard chow.

Mice were dosed intraperitoneally with kynurenine (100 mg kg^−1^; HY‐104026, MCE) every other day from day 0 through day 35; matched controls received PBS at the corresponding time points.

For in vivo TNFR2 neutralization, mice were randomly assigned to Control + Isotype, Stress + Isotype, Control + TNFR2Ab, and Stress + TNFR2Ab groups (*n* = 10 per group). An anti‐mouse TNFR2 blocking antibody (Leinco Technologies, T254) was administered intraperitoneally at 100 µg/20 g body weight every 2 d starting 1 d before tumor cell inoculation. Armenian hamster IgG isotype control antibody (Leinco Technologies, I‐140) was given at the same dose and schedule.

#### 4.4.2 Behavioral Tests

Depression‐related behavior was characterized by SPT, FST, and TST. For the SPT, animals were housed singly and allowed simultaneous access to water and a 2% sucrose solution for 12 h, with the two bottles exchanged at 6 h to reduce positional bias. Preference for sucrose was calculated as 100 × sucrose intake/(sucrose intake + water intake).

The FST was conducted in cylinders measuring 15 cm in diameter and 40 cm in height, with water maintained at 25 ± 1 °C to a depth of 25 cm. Each session lasted 6 min. The initial 2 min were excluded as acclimation, and immobility during the remaining 4 min was determined from video recordings by an investigator blinded to group identity.

For the TST, adhesive tape was attached 1 cm from the tail tip, and animals were suspended 50 cm above the surface for 6 min. Immobility was scored during the final 4 min after excluding the initial 2‐min acclimation period, with group identity concealed from the evaluator.

#### 4.4.3 Bone Marrow‐Derived Macrophage (BMDM) Preparation

Bone marrow harvested from the femurs and tibias of 6–8‐week‐old C57BL/6 mice was processed into a single‐cell suspension by flushing and 100‐µm filtration. After a 5 min centrifugation at 800 rpm, the cell fraction was maintained for 7 d in M‐CSF‐containing DMEM (20 ng mL^−1^; RP01216S, ABclonal) for macrophage differentiation.

#### 4.4.4 Cell Functional Assays

Cell migration was assessed using Transwell assays with 20% FBS in the lower chamber; after 24 h, migrated cells were fixed, stained, and quantified in five randomly selected fields. For colony formation, 1 × 10^3 cells were plated per well in 6‐well plates, cultured for 10 d, and colonies were fixed, stained with crystal violet, and counted.

#### 4.4.5 In Vitro Pharmacological and Antibody Rescue Experiments

BMDMs or RAW264.7 cells were allocated to Vehicle, Kyn, inhibitor/antibody alone, or combination treatment groups. Treatment concentrations were 200 µm for Kyn, 12.5 µg mL^−1^ for the TNFR2‐neutralizing antibody (Sino Biological, 50128‐RN204), 10 µm for ST101 (ProbeChem, PC‐49350), and 1 µm for SR1. Where indicated, Kyn and the corresponding inhibitor or antibody were added simultaneously and maintained for 48 h. Each condition was tested in three replicate wells, after which cells were harvested for phenotypic analyses.

#### 4.4.6 ELISA

ELISA quantification included Kyn in plasma, feces, lung tumor tissues, and bacterial culture supernatants (FineTest, EU2565); cortisol and norepinephrine in plasma (FineTest, EM1721 and EM1862, respectively); and IL‐10 and TGF‐β in plasma and cell culture supernatants (ABclonal, RK00016 and RK00057, respectively). Assays were performed following the specifications supplied with the respective kits.

#### 4.4.7 Western Blotting

Cellular or tissue proteins were extracted in inhibitor‐supplemented RIPA buffer and normalized by BCA assay. Equivalent protein inputs were subjected to SDS‐PAGE and PVDF transfer, followed by antibody‐based detection after membrane blocking. Chemiluminescent signals were recorded for analysis. Details of the antibodies used are provided in Supplementary Table S2.

#### 4.4.8 Quantitative RT‐PCR

RNA‐to‐cDNA preparation and SYBR‐based amplification were performed with commercial kits (AG21023 and AG11728, Accurate Biology; 11184ES08, Yeasen) using a LightCycler 96 platform. Relative transcript abundance was normalized to GAPDH and calculated by the 2^−ΔΔCt method. Primer sequences are listed in Table S1.

#### 4.4.9 Fecal DNA Extraction and Quantitative PCR

DNA was isolated from 10 mg of freshly collected feces using the TIANamp Stool DNA Kit (TIANGEN, Beijing, China). Gene‐specific qPCR was subsequently performed using Taq Pro Universal SYBR qPCR Master Mix (11184ES08, Yeasen) on a LightCycler 96 system (Roche, Basel, Switzerland). Relative target abundance was normalized to 16S rRNA and calculated using the 2^−ΔΔCt method. Absolute *L. reuteri* abundance was determined from plasmid‐based calibration curves prepared by 10‐fold serial dilution of the target sequence. Results were reported as bacterial copy number per gram of fecal material. Primer information is listed in Table S1.

#### 4.4.10 Flow Cytometry

Tumor and spleen tissues were processed into single‐cell suspensions, with tumor tissues enzymatically dissociated using collagenase IV (3 mg mL^−1^) and DNase I (10 µg mL^−1^). After viability staining and Fc blocking, cells were labeled with the indicated surface or intracellular antibodies as appropriate. Samples were analyzed on a CytoFLEX flow cytometer (Beckman Coulter), with downstream analysis performed in FlowJo. Gating strategies and antibody details are provided in Figure S7 and Table S3, respectively.

#### 4.4.11 Immunohistochemistry and Immunofluorescence

Paraffin‐embedded lung sections were processed for antigen retrieval and subjected to either chromogenic or fluorescence‐based immunostaining. Arg1 IHC was performed using anti‐Arg1 antibody (1:500; 16001‐1‐AP, Proteintech) with HRP/DAB detection and hematoxylin counterstaining. Immunofluorescence staining used the indicated primary and fluorophore‐conjugated secondary antibodies with DAPI nuclear labeling. Images were acquired by conventional or confocal microscopy as appropriate. Antibody details are provided in Table S2.

#### 4.4.12 Single‐Cell RNA Sequencing

Single‐cell suspensions prepared from tumor tissues were subjected to 10x Genomics‐based scRNA‐seq, followed by paired‐end 150‐bp sequencing on an Illumina NovaSeq 6000 at a target depth of >50 000 reads per cell. Raw data were processed with Cell Ranger v8.0.1 and analyzed in Seurat. Cells with 200–2,500 detected genes, <20% mitochondrial transcripts, and <10,000 UMI counts were retained. After normalization, the 2,000 most variable genes were used for principal component analysis, and batch effects were corrected with Harmony using the first 20 principal components. Cell clusters were visualized by UMAP and annotated using SingleR together with canonical marker genes.

#### 4.4.13 16S rRNA Gene Sequencing and Microbiota Analysis

Fecal microbiota profiling was performed by Majorbio Bio‐Pharm Technology Co., Ltd. (Shanghai, China). The V3–V4 region of the bacterial 16S rRNA gene was sequenced on an Illumina NextSeq 2000 platform. Sequence processing and quality control were conducted in QIIME, and taxonomic annotation was based on the Greengenes database. OTUs were clustered at 97% sequence similarity using UPARSE v7.1. PCoA and LEfSe analyses were performed on the Majorbio Cloud platform; LEfSe significance was defined as Kruskal–Wallis *P* ≤ 0.05, LDA score >2, and FDR <0.05.

#### 4.4.14 Bulk RNA Sequencing

Bulk RNA sequencing was performed by the sequencing service provider following standard RNA quality‐control and library‐preparation procedures. Libraries were generated using the NEBNext Ultra RNA Library Prep Kit for Illumina and sequenced on an Illumina HiSeq platform. Differential expression and downstream functional analyses were performed using the corresponding bioinformatic workflow.

#### 4.4.15 Metabolomics

Untargeted plasma metabolomics was conducted by Majorbio Bio‐Pharm Technology Co., Ltd. (Shanghai, China) on a Thermo UHPLC‐Q Exactive system. Plasma metabolites were extracted with methanol before LC‐MS analysis according to the service provider's standard protocol. Metabolites were annotated against HMDB and Metlin, followed by KEGG‐based pathway enrichment analysis.

#### 4.4.16 Targeted Quantification of Tryptophan Metabolites

Targeted plasma tryptophan metabolite profiling was performed by Majorbio Bio‐Pharm Technology Co., Ltd. (Shanghai, China) using LC‐MS/MS on a Nexera LC‐40–QTRAP 6500+ platform. Sample extraction, chromatographic separation, and mass spectrometric acquisition followed the provider's standardized workflow. The same analytical platform was applied to plasma samples from 41 patients with gastric cancer. Patients were divided into HAMD‐low and HAMD‐high groups according to the median HAMD score. Kynurenine‐pathway activity was represented by the plasma Kyn/Trp ratio, calculated as kynurenine concentration divided by tryptophan concentration.

### 4.5 Quantification and Statistical Analysis

Analyses were conducted in GraphPad Prism 9. Sample sizes and statistical tests are specified in the figure legends. Two‐tailed unpaired Student's t tests were used for two‐group comparisons, and one‐ or two‐way ANOVA with appropriate multiple comparisons for multi‐group or multifactor analyses. *P* < 0.05 was regarded as significant. Survival was assessed by Kaplan–Meier curves and log‐rank tests. Mice meeting predefined humane endpoints, including substantial weight loss or severe distress, were euthanized.

## Author Contributions

Sicheng Zhao and Zhiyuan Song conducted the statistical analyses, interpreted the results, and prepared the manuscript, with input from all authors. Jingcheng Zhang and Jiaheng Lou analyzed the transcriptomic and metabolomic datasets. Sicheng Zhao, Zhiyuan Song, Fengmei Qiu, and Shuo Zhang designed the experiments and contributed to manuscript drafting. Sicheng Zhao, Yunhai Wei, and Zhiyuan Song supervised and performed sample and data collection. Jingcheng Zhang, Jiaheng Lou, Boqiang Liu, and Tao Jiang reviewed and verified the underlying data. Guangji Zhang contributed to the conceptualization of the study, funding acquisition, resource provision, and project administration. All authors have read and approved the final version of the manuscript.

## Conflicts of Interest

The authors declare no conflicts of interest.

## Data and Code Availability

The datasets generated and analyzed during the current study are available from the corresponding author upon reasonable request. Sequencing data will be deposited in a public repository upon acceptance of the manuscript.

## Supporting information




**Supporting File 1**: advs77612‐sup‐0001‐SuppMat.docx.

**Supporting File 2**: advs77612‐sup‐0002‐Tables.xlsx.

## Data Availability

The data that support the findings of this study are available from the corresponding author upon reasonable request.
