# Supplementary material for: Chronic Stress Promotes Gastric Cancer Metastasis by Inducing Microbiota‐Associated Kynurenine Accumulation and Macrophage Remodeling
Source: Adv Sci (Weinh). 2026 Sep 3:e77612. Online ahead of print. doi: 10.1002/advs.77612 (PMC13542406; doi:10.1002/advs.77612)
Supplement: Supplementary file 1 [file ADVS-9999-e77612-s001.docx]

**Supplementary Data**

**Supplementary information includes:**

**Supplementary Tables:**

**Table S1.** Sequences of the qPCR primers.

**Table S2** Antibodies used for WB, IF, and IHC.

**Table S3** Antibodies used for flow cytometry.

**Table S4**. Baseline characteristics of the clinical cohorts used for Figure 7.

• Table S4A. Baseline characteristics of the survival cohort related to Figure 7A.

• Table S4B. Baseline characteristics of the fecal microbiota cohort related to Figure 7B.

• Table S4C. Baseline characteristics of the plasma cohort related to Figure 7C.

• Table S4D. Baseline characteristics of the tumor tissue cohort related to Figure 7D.

**Supplementary Figures:**

**Figure S1**. Physiological indices and subcutaneous tumor modeling under chronic restraint stress.

**Figure S2**. Additional scRNA-seq and immunologic validation of stress-associated macrophage programs.

**Figure S3**. Additional validation of stress-induced gut microbial dysbiosis and microbiota transfer.

**Figure S4. Additional validation of** L. reuteri **colonization, tryptophan restriction, and myeloid profiling.**

**Figure S5. Validation experiments linking microbiota-associated kynurenine to macrophage phenotype remodeling.**

**Figure S6**. Additional evidence supporting TNFR2–C/EBPβ signaling in Kyn-induced macrophage phenotype remodeling.

**Figure S7. Gating strategy for tumor-infiltrating myeloid and lymphoid cell populations.**

### Supplementary Table 1. Sequences of the qPCR primers.

| Gene | Forward | Reverse |
| --- | --- | --- |
| *Arg1* | TGTCCCTAATGACAGCTCCTT | GCATCCACCCAAATGACACAT |
| *Mrc1* | GCTTCCGTCACCCTGTATGC | TCATCCGTGGTTCCATAGACC |
| *Tgfb* | CCACCTGCAAGACCATCGAC | CTGGCGAGCCTTAGTTTGGAC |
| *Il10* | GCTGGACAACATACTGCTAACC | ATTTCCGATAAGGCTTGGCAA |
| *Nos2* | CAAGCACCTTGGAAGAGGAG | AAGGCCAAACACAGCATACC |
| *Tnfa* | TCGTAGCAAACCACCAAGTG | AGATAGCAAATCGGCTGACG |
| *Il1b* | GAAATGCCACCTTTTGACAGTG | TGGATGCTCTCATCAGGACAG |
| *Il6* | GCCACCGTTACCCTGATTTG | TCCTGTGGTAGTCCATTCTCTG |
| *Gapdh* | CTGGCATTGCTCTCAATGAC | CCGTATTCATTGTCATACCAGG |
| *L. reuteri* | TTGGAAATGTTCCACAAGAC | TTGTGAGTTTGGATTGAACC |
| 16S rRNA | CCTACGGGNGGCWGCAG | GACTACHVGGGTATCTAATCC |

### Supplementary Table 2. Antibodies used for WB, IF, and IHC.

| Antibody | Company | Catalog No. |
| --- | --- | --- |
| IDO1 | Proteintech Group | 66528-1-Ig |
| C/EBPβ | CST | 3087 |
| Phospho-C/EBPβ | CST | 3084 |
| p65 | CST | 8242 |
| Phospho-p65 | ABclonal | AP0124 |
| IκB | CST | 4812 |
| Phospho-IκB | ABclonal | AP0707 |
| β-Actin | ABclonal | AC026 |
| GAPDH | ABclonal | AC033 |
| F4/80 | HaoKebio | B06001R |
| CD86 | HaoKebio | B03019R |
| CD206 | HaoKebio | B03006R |
| Occludin | Abcam | ab216327 |
| Claudin1 | Abcam | ab180158 |
| ZO-1 | Abcam | ab216880 |

### Supplementary Table 3. Antibodies used for flow cytometry.

| Antibody | Company | Catalog No. |
| --- | --- | --- |
| Fixable Viability Stain 700 | BD | 564997 |
| APC-Cy™7 Rat Anti-Mouse CD45 | BD | 557659 |
| FITC Rat Anti-CD11b | BD | 557396 |
| RY610 Rat Anti-Mouse F4/80 | BD | 759374 |
| Alexa Fluor™ 647 Rat Anti-Mouse CD206 | BD | 568809 |
| BV421 Rat Anti-Mouse CD86 | BD | 564198 |
| PerCP-Cy™5.5 Rat Anti-Mouse CD45 | BD | 550994 |
| APC-Cy™7 Hamster Anti-Mouse CD3e | BD | 557596 |
| BV510 Rat Anti-Mouse CD4 | BD | 563106 |
| FITC Rat Anti-Mouse CD8a | BD | 553030 |
| BV421 Rat Anti-Mouse CD25 | BD | 562606 |
| Alexa Fluor® 647 Rat anti-Mouse Foxp3 | BD | 560401 |
| PE-Cy™7 Mouse Anti-Mouse NK-1.1 | BD | 552878 |
| Alexa Fluor^®^ 700 Rat Anti-Mouse Ly-6G | BD | 561236 |
| Brilliant Violet 510™ anti-mouse Ly-6C | Biolegend | 128033 |

**Figure S1. Physiological indices and subcutaneous tumor modeling under chronic restraint stress.**

(A, B) Longitudinal body-weight trajectories in the lung metastasis (A) and peritoneal metastasis (B) experiments under Control and Stress conditions.

(C, D) Plasma cortisol (C) and norepinephrine (D) concentrations (n = 6).

(E) Experimental scheme for the CRS-based subcutaneous tumor model.

(F, G) Gross appearance of subcutaneous tumors (F) and corresponding tumor weights (G) (n = 8).

Values are shown as mean ± SD. **P < 0.01, ****P < 0.0001; ns, not significant. Statistical tests: two-way ANOVA for A and B; two-tailed unpaired Student’s t tests for C, D, and G.

**Figure S2. Additional scRNA-seq and immunologic validation of stress-associated macrophage programs.**

**(A) Canonical marker expression used for annotation of major cell populations in lung metastatic lesions.**

**(B) Relative abundance of major cell populations in Control and Stress groups.**

**(C) Macrophage subset marker-gene expression heatmap.**

**(D) M1- and M2-associated transcriptional signature distributions across macrophages.**

**(E) mRNA levels of macrophage polarization- and inflammation-related genes (n = 3).**

**(F) ARG1 immunohistochemistry in lung metastatic lesions with average optical density (AOD) quantification (n = 5).**

**(G, H) Plasma IL-10 (G) and TGFβ1 (H) concentrations (n = 3).**

**(I) Flow-cytometric assessment of CD11b⁺F4/80⁺ macrophages in spleen and tumor tissues.**

**(J) Frequencies of CD4⁺ T cells, CD8⁺ T cells, NK cells, and Treg cells in tumor tissues (n = 5).**

**Values are shown as mean ± SD. *P < 0.05, **P < 0.01; ns, not significant. Statistical tests: two-tailed unpaired Student’s t tests for E–J.**

**Figure S3. Additional validation of stress-induced gut microbial dysbiosis and microbiota transfer.**

**(A) PCoA of fecal microbial communities in Control and Stress mice.**

**(B) ACE diversity index (n = 5).**

**(C) Microbial dysbiosis index (MDI) (n = 5).**

**(D) Shared and group-specific OTUs.**

**(E, F) Family- (E) and genus-level (F) microbial composition.**

**(G) Fecal bacterial culture before and after antibiotic treatment.**

**(H) PCoA of recipient microbiota following Control-FMT or Stress-FMT.**

**(I) MDI in Control-FMT and Stress-FMT recipients (n = 5).**

**(J) Plasma IL-10 concentrations after FMT (n = 5).**

**(K) Fecal bacterial DNA content in Control+ABX and Stress+ABX mice on days 0 and 14 (n = 4).**

**(L) PCoA of fecal microbiota in Control, Stress, and co-housed mice.**

**(M) ACE diversity index across Control, Stress, and co-housed groups (n = 5).**

**(N) Body-weight profiles of Control, Stress, and co-housed mice (n = 6).**

**Values are shown as mean ± SD. *P < 0.05, ***P < 0.001, ****P < 0.0001; ns, not significant. Statistical tests: two-tailed unpaired Student’s t tests for B, C, I, and J; two-way ANOVA with Sidak’s multiple-comparisons test for K; one-way ANOVA with Tukey’s multiple-comparisons test for M, N.**

**Figure S4. Additional validation of L. reuteri colonization, tryptophan restriction, and myeloid profiling.**

**(A) Relative abundance of *L. reuteri* in fecal samples from Control and Stress groups (n = 3).
(B) Body weight changes during oral gavage with Vehicle or *L. reuteri*.
(C) Absolute quantification of fecal *L. reuter*i abundance in Vehicle- and *L. reuteri*-treated mice (n = 4).
(D) Representative immunofluorescence images of lung metastatic lesions (F4/80, CD86, CD206, DAPI; scale bar = 50 μm).
(E) Partial least squares-discriminant analysis (PLS-DA) of plasma metabolomic profiles in Control and Stress groups.
(F) Experimental scheme illustrating oral gavage of *L. reuteri* under a tryptophan-deficient diet.
(G) Experimental scheme illustrating the tryptophan-deficient diet intervention in Control and Stress mice.
(H) Experimental scheme for oral gavage of *L. reuteri* in the subcutaneous tumor model.
(I) Tumor growth curves of subcutaneous tumors in Vehicle- and *L. reuteri*-treated mice.
(J) Representative gross images of subcutaneous tumors and quantification of tumor weight in Vehicle- and *L. reuteri*-treated mice (n = 5).
(K, L) Flow cytometric analysis (K) and quantification (L) of immune cell populations in pulmonary metastases and subcutaneous tumors (n = 4).
Data are presented as the mean ± SD. *P < 0.05; **P < 0.01; ***P < 0.001; ns, not significant. (A, C, J, two-tailed unpaired Student’s t test; B, I, two-way ANOVA followed by Sidak’s multiple comparisons test; L, multiple t tests.)**

**Figure S5. Validation experiments linking microbiota-associated kynurenine to macrophage phenotype remodeling.**

**(A) PCA of targeted plasma metabolomic profiles from Vehicle- and *L. reuteri*-treated mice.**

**(B, C) Kyn concentrations in feces (B) and tumor tissues (C) from Control and Stress mice (n = 3).**

**(D, E) Spearman correlations Kyn levels and lung metastatic burden in the Stress versus Control model (D) and the *L. reuteri* versus Vehicle model (E).**

**(F) Design of the bacterial and fecal culture experiments used to assess Kyn production.**

**(G, H) Fecal (G) and tumor-tissue (H) Kyn concentrations in Control+ABX and Stress+ABX mice (n = 3).**

**(I) Overview of the tryptophan–kynurenine metabolic pathway.**

**(J) Occludin and claudin1 protein expression in colon tissues after Vehicle or *L. reuteri* treatment.**

**(K) Colony formation of HGC-27 and AGS cells across the indicated Kyn concentrations, with colony counts (n = 3).**

**(L) Transwell migration of HGC-27 and AGS cells across the indicated Kyn concentrations, with migrated-cell counts (n = 3).**

**(M, N) M2-associated gene expression and CD206⁺ macrophage frequency in Kyn-treated BMDMs (M) and RAW264.7 cells (N) (n = 3).**

**Values are shown as mean ± SD. *P < 0.05, **P < 0.01, ****P < 0.0001; ns, not significant. Statistical tests: two-tailed unpaired Student’s t tests for B, C, G, and H; one-way ANOVA with multiple-comparison correction for K–N. For K–N, significance was assessed relative to the 0 μM Kyn group.**

**Figure S6. Additional evidence supporting TNFR2–C/EBPβ signaling in Kyn-induced macrophage phenotype remodeling.**

(A) GSEA of the core AHR-response signature in BMDMs exposed to 200 μM Kyn for 6 h versus vehicle.

(B) CD206 MFI in BMDMs under the indicated treatments (n = 3).

(C) AHR target-gene expression in BMDMs under the indicated treatments.

(D) KEGG enrichment of differentially expressed genes from scRNA-seq-derived macrophages in Control versus Stress mice, highlighting TNF signaling.

(E) Working model for Kyn-associated macrophage remodeling through TNFR2–C/EBPβ signaling.

(F, G) p-C/EBPβ and total C/EBPβ protein levels in tumor tissues, with p-C/EBPβ/total C/EBPβ quantification (n = 3).

(H) p-C/EBPβ and total C/EBPβ levels in metastatic lung tissues after microbiota manipulation (H₂O versus ABX; single housing versus co-housing), with corresponding p-C/EBPβ/total C/EBPβ ratios (n = 3).

(I) C/EBPβ localization in F4/80⁺ macrophages within lung metastatic lesions under the indicated microbiota conditions; nuclear/cytoplasmic C/EBPβ ratios are shown below (n = 3; scale bar = 50 μm).

(J) M2-associated gene expression in BMDMs (n = 3).

(K) IL-10 and TGFβ concentrations in BMDM culture supernatants (n = 3).

(L) CD206 MFI in BMDMs after the indicated treatments (n = 3).

(M) Design of the TNFR2-neutralization experiment in the CRS metastasis model.

Values are shown as mean ± SD. *P < 0.05, **P < 0.01, ****P < 0.0001; ns, not significant. Statistical tests: one-way ANOVA with multiple-comparisons correction for B, C, J, K, and L; two-tailed unpaired Student’s t test for F and G; two-way ANOVA with Sidak’s multiple-comparisons test for H and I. Asterisks in C indicate comparisons with the FICZ-treated group, whereas those in J indicate comparisons with the Kyn-treated group.

 **Figure S7. Gating strategy for tumor-infiltrating myeloid and lymphoid cell populations.**
